# Impact of Iron (II) Chloride Treatment on the Physical and Metabolic Changes in Mungbean Sprouts

**DOI:** 10.1002/fsn3.71558

**Published:** 2026-02-24

**Authors:** Aerin Park, Byeong Cheol Kim, Sung Don Lim, Sung Hoon Park, Jungmin Ha

**Affiliations:** ^1^ Department of Wellness Bio Industry Gangneung‐Wonju National University Gangneung Republic of Korea; ^2^ Department of Plant Science Gangneung‐Wonju National University Gangneung Republic of Korea; ^3^ Molecular Plant Physiology Laboratory, Department of Applied Plant Sciences, Graduate School Sangji University Wonju Republic of Korea; ^4^ Department of Food & Nutrition Gangneung‐Wonju National University Gangneung Republic of Korea; ^5^ Haeram Institute of Bakery Science Gangneung‐Wonju National University Gangneung Republic of Korea; ^6^ Department of Agriculture, Forestry and Bioresources and Research Institute of Agriculture and Life Sciences Seoul National University Seoul Republic of Korea; ^7^ Crop Genomics Laboratory, Plant Genomics and Breeding Institute Seoul National University Seoul Republic of Korea

**Keywords:** environmental stress, isoflavones, mineral treatment, mungbean sprouts, seed coat color, ultra‐performance liquid chromatography (UPLC)

## Abstract

Legume crops are rich in carbohydrates, proteins, and various secondary metabolites such as isoflavones. Micronutrient treatments regulate plant biological activities, including stomatal function, hormone balance, and antioxidant accumulation, thereby improving resistance to environmental stress. This study investigated the effect of FeCl_2_ treatment, which changed the seed coat color, on the physical and biochemical characteristics of mungbean sprouts. After 72 h of cultivation with FeCl_2_ (Fe–72 h), no significant differences in physical traits were observed compared with the control. However, the levels of isoflavone aglycones (daidzein, genistein, and glycitein) were significantly higher. These findings suggest that FeCl_2_ treatment does not affect the physical quality of mungbean sprouts but enhances their metabolic quality by catalyzing isoflavone biosynthesis, as these compounds function as iron chelators. Tissue‐specific analysis revealed that the seed coat and cotyledons accumulated the highest levels of isoflavone aglycones. The seed coat and cotyledons are directly exposed to FeCl_2_ during soaking, whereas newly developing tissues, such as the hypocotyl, are only indirectly affected. This difference in mineral exposure among tissues likely influences the biosynthesis of secondary metabolites. The observed increase in isoflavone content may improve stress resistance during mungbean cultivation and enhance the nutritional quality of mungbean sprouts as a health‐promoting food.

## Graphical abstract

FeCl_2_ treatment darkened the seed coat color of mungbean sprouts without significantly affecting physical traits compared with the control (CK). In contrast, isoflavone aglycone contents were markedly increased, particularly in the seed coat and cotyledon. These results indicate that FeCl_2_ treatment enhances the metabolic quality of mungbean sprouts without compromising physical quality.

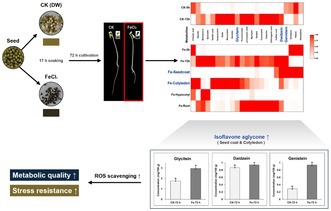

## 1 Introduction

Mungbean ( *Vigna radiata* L.) is a representative legume crop widely cultivated and consumed in Asia. It is an important food resource because of its high carbohydrate (50%–60%) and protein (20%–24%) contents, along with various vitamins and minerals (Hou et al. 2019; Tang et al. 2014). In addition, mungbean contributes to soil fertility through biological nitrogen fixation and exhibits high water‐use efficiency, allowing stable growth under relatively dry environmental conditions. Mungbeans are commonly consumed in the form of sprouts during the early stages of germination, during which enhanced metabolic activity leads to improved nutritional and functional value.

Secondary metabolites are not directly essential for plant growth; however, they play crucial roles in plant adaptation and defense against environmental stresses. Mungbean sprouts contain a wide range of secondary metabolites, including phenolic compounds and isoflavones. In particular, isoflavones such as daidzein, genistein, and glycitein are legume‐specific metabolites, and their contents have been reported to increase significantly during germination and sprout cultivation.

Environmental stresses are major limiting factors affecting plant growth and productivity, often disrupting water and mineral transport, cellular redox homeostasis, and metabolic balance. Among these stresses, drought has recently emerged as a particularly critical constraint because of its increasing frequency and severity, leading to pronounced mineral imbalance and oxidative stress in plant tissues (Singh et al. 2021; Iqbal et al. 2020).

To mitigate the adverse effects of environmental stresses, mineral‐based treatments have received growing attention. Micronutrients such as zinc (Zn), manganese (Mn), and iron (Fe) are known to play important roles in plant stress regulation. Zn and Mn are primarily associated with hormonal regulation, maintenance of stomatal function, and antioxidant protection, and their beneficial effects on growth characteristics and storage stability of legume sprouts have been demonstrated in previous studies (Waraich et al. 2011; Hebbern et al. 2009; Jin et al. 2022).

Among these micronutrients, iron (Fe) plays a particularly important role because of its direct involvement in redox reactions, making it closely associated with the regulation of reactive oxygen species (ROS) and antioxidant metabolism, as well as the maintenance of legume‐specific physiological functions such as nitrogen fixation (Rai et al. 2021; Tripathi et al. 2018). Previous studies have reported that soaking soybean ( *Glycine max* ) seeds in iron chloride (FeCl_2_) solution can influence antioxidant activity and flavonoid composition during germination, depending on germination conditions (Wleklik et al. 2023).

In addition, iron has been implicated in the regulation of secondary metabolic pathways, including the phenylpropanoid pathway, and alterations in iron homeostasis may affect the accumulation patterns of specific secondary metabolites (Jan et al. 2021; Salam et al. 2023; Yang et al. 2018). However, studies that systematically investigate the effects of iron treatment on physical traits and secondary metabolite accumulation in mungbean sprouts remain limited.

Therefore, in this study, FeCl_2_, MnCl_2_, and ZnCl_2_ were applied during the seed soaking process to comparatively evaluate their effects on the physical and biochemical characteristics of mungbean sprouts after germination, with a particular focus on the role of iron in modulating sprout quality and secondary metabolite profiles.

## 2 Materials and Methods

### 2.1 Sample Preparation

Mungbean ( *Vigna radiata* L.) seeds of the variety “Sanpo” were cultivated and harvested at the Gangneung‐Wonju National University Experimental Farm, Gangneung, South Korea (37.77° N, 128.86° E). Seeds were washed three times with distilled water. For each treatment, 60 seeds were soaked for 17 h at 37°C in 25 mL of either distilled water (control) or 5 mM FeCl_2_, MnCl_2_, or ZnCl_2_ solutions in an incubator (JEIO TECH, ISS‐4075R, Daejeon, Korea) (Jin et al. 2022; Zhao et al. 2020).

The concentrations of FeCl_2_, MnCl_2_, and ZnCl_2_ were set at 5 mM on the basis of previous studies reporting that mineral salt treatments within the low millimolar range can induce physiological and metabolic responses in legume seeds or sprouts without causing severe phytotoxic effects (Jin et al. 2022; Zhao et al. 2020). On the basis of these reports, preliminary concentration tests were conducted prior to the main experiment, which showed that treatment at 10 mM resulted in reduced sprout length, whereas treatment at 5 mM did not cause abnormal growth or visible phytotoxic symptoms and was therefore considered appropriate for the present study (data not shown).

After soaking, the seeds were germinated at 28°C ± 2°C for 3 days using a sprout cultivator (Sundotcom, ST001A, Seoul, Korea), under complete dark conditions, with water sprayed for 2 min every 4 h (Kim et al. 2021).

After cultivation, soaked seeds (0 h) and sprouts cultivated (72 h) were rapidly frozen in liquid nitrogen and stored in a deep freezer for 5 days. Samples were then freeze‐dried for 5 days at −80°C using a freeze dryer (ilShin Biobase, FD 8508, Gyeonggi, Korea), ground into fine powder, and used for antioxidant capacity and secondary metabolite analyses.

Sprout extracts were prepared with 70% ethanol (v/v; Supelco, Cat. No. 1009831011, Germany) at 0.1 g/mL (w/v). Extract concentrations were adjusted according to each assay: 20 mg/mL for total flavonoids, 10 mg/mL for total phenols and DPPH radical scavenging activity, and 1 mg/mL for ABTS radical scavenging activity.

### 2.2 Measurement of Physical Traits

Fifty mungbean sprouts were randomly selected for measurement. Tissue lengths were determined using ImageJ software (version 1.54; public domain; Schneider et al. 2012). A reference line of 1 cm was set, and lengths were measured using the freehand line tool. Cotyledon length was measured from the hypocotyl boundary to the tip, hypocotyl length from the cotyledon to the point where the thickness of the hypocotyl begins to decrease, and root length from the end of the hypocotyl measurement point to the root tip. Hypocotyl thickness was measured at its widest point using a straight line.

For fresh and dry weight measurements, the same 50 sprouts were gently blotted dry and combined into a single pooled sample to obtain an integrated biomass measurement. Fresh weight was measured using a precision balance (OHAUS, PX223KR, USA). The pooled sample was then freeze‐dried, and its dry weight was measured. The fresh‐to‐dry weight ratio was calculated on the basis of the pooled fresh and dry weight measurements.

Seed coat color was determined using RGB values obtained from 50 seed coats, and the mean values of each color component were calculated.

### 2.3 Quantification of Total Phenolic and Flavonoid Content

The total phenolic content was measured using a modified method of Lee and Lee (2008), with gallic acid (Sigma‐Aldrich, G7384, USA) as the standard (0, 50, 100, 200 mg/L). Each extract (or standard, 100 μL) was mixed with 50 μL of Folin–Ciocalteu reagent (Sigma‐Aldrich, Cat. No. 47641, USA) and allowed to react for 5 min. Then, 300 μL of 20% Na_2_CO_3_ solution (FUJIFILM, 199–01605, Tokyo, Japan) was added, and the mixture was incubated for 15 min at 25°C in the dark. Afterward, 1000 μL of distilled water was added, and the solution was centrifuged for 2 min (GYROZEN, 1730R, Seoul, Korea). The supernatant was transferred into a 96‐well plate (200 μL per well), and absorbance was measured at 740 nm using a spectrophotometer (Thermo Scientific, 51,119,000, USA).

The total flavonoid content was measured using a modified method of Matejić et al. (2013), with quercetin (Sigma‐Aldrich, Q4951, USA) as the standard (0, 50, 100, and 200 mg/L). A reagent mixture of 1 M potassium acetate (FUJIFILM, 169–21,965, Tokyo, Japan) and 10% aluminum nitrate nonahydrate (JUNSEI, 37350–1201, Tokyo, Japan) was prepared at a 1:1 (v/v) ratio. Then, 160 μL of this mixture was added to 400 μL of quercetin standard or extract solution and incubated for 30 min at 25°C in the dark. The solution was centrifuged for 2 min, and the supernatant was transferred into a 96‐well plate (150 μL per well). Absorbance was measured at 420 nm using a spectrophotometer.

### 2.4 Measurement of Antioxidant Capacity

The DPPH radical scavenging activity was measured using the OxiTec DPPH Antioxidant Assay Kit (BIOMAX, BO‐DPH‐500, Gyeonggi, Korea), following the manufacturer's protocol. Trolox (0, 20, 40, 60, and 100 mg/L) was used as the standard. Extract or Trolox (20 μL each) was added to a 96‐well plate. For the blank wells, 70% ethanol (20 μL) was added to blank wells 1 and 2, and 100% ethanol (20 μL) was added to blank well 3. Subsequently, 80 μL of assay buffer was added to all wells, and 100 μL of 100% ethanol was added to blank wells 2 and 3. Finally, 100 μL of DPPH solution was added to the wells containing extracts, Trolox, and blank well 1. The reaction mixture was incubated for 30 min at 25°C in the dark, after which absorbance was measured at 520 nm using a spectrophotometer.

The ABTS radical scavenging activity was measured with slight modifications to the method of Kusumah et al. (2020). Ascorbic acid (FUJIFILM, Cat. No. 012–04802, Tokyo, Japan) was used as the standard (0, 5, 10, 20, and 40 mg/L). A solution of 7.4 mM ABTS (Roche, Cat. No. 10102946001, Mannheim, Germany) and 2.6 mM potassium persulfate (YAKURI, Cat. No. 28718, Kyoto, Japan) was prepared at a 1:1 (v/v) ratio and stored in the dark at 25°C for 24 h. The mixture was then diluted with 10 mM PBS buffer (Gibco, Cat. No. 10010023, USA) to reach an absorbance of 0.7 ± 0.03 at 740 nm. Extracts or ascorbic acid (20 μL) were added to a 96‐well plate, followed by 180 μL of the ABTS mixture. The mixture was then incubated for 10 min at 25°C in the dark, and absorbance was measured at 740 nm using a spectrophotometer.

### 2.5 Quantification of Secondary Metabolites Using Ultra‐Performance Liquid Chromatography (UPLC)

Secondary metabolites in mungbean sprouts were quantified using a Nexera UPLC system (Shimadzu, Kyoto, Japan). Separation and analysis were performed using a ZORBAX SB‐C18 column (3.5 μm, 4.6 × 150 mm; Agilent, PN 863953–902, Santa Clara, USA). The column oven was maintained at 40°C, and 2 μL of each sample was injected. The mobile phases comprised ultrapure water with 0.1% acetic acid (Fisher, W5‐4, Seoul, Korea; FUJIFILM, 017–00256, Tokyo, Japan) (solvent A) and acetonitrile (Fisher, A998‐4, Seoul, Korea) (solvent B).

The flow rate was set at 1 mL/min under the following gradient conditions: 0–10 min (95% A), 10–11 min (95%–90% A), 11–20 min (90% A), 20.1–22 min (80% A), 22–23 min (80%–90% A), 23.1–24 min (95% A), 24–25 min (95%–65% A), 25–29 min (65% A), 29–32 min (65%–50% A), 32–35 min (50% A), 35.1–40 min (95% A). The standard substances used were 4‐hydroxybenzoic acid, biochanin A, caffeic acid, catechin, chlorogenic acid, cinnamic acid, coumestrol, daidzein, daidzin, epicatechin, formononetin, gallic acid, genistein, genistin, glycitein, glycitin, isoquercitrin, isovitexin, kaempferol, myricetin, neochlorogenic acid, *p*‐coumaric acid, protocatechuic acid, quercetin, resveratrol, rutin, sinapic acid, syringic acid, *t*‐ferulic acid, vanillic acid, and vitexin (Chemfaces, Wuhan, China). Calibration curves for all the standard substances were prepared at concentrations of 15–90 mg/L (Supplementary Figure S1). Absorbance was measured using a photodiode array detector across a wavelength range of 190–800 nm, and limits of detection (LOD) and limits of quantification (LOQ) were determined for each compound.

### 2.6 Statistical Analysis

All experiments, except for fresh and dry weight measurements, were performed in triplicate, and results are expressed as mean ± standard deviation (SD). Statistical significance was assessed using one‐way analysis of variance, followed by Duncan's multiple range test for post hoc comparisons. Differences were considered significant at *p* < 0.05 (Ståhle and Wold 1989). Fresh weight, dry weight, and fresh‐to‐dry weight ratio were determined using a single pooled sample consisting of 50 sprouts per treatment. Because of this experimental design, statistical analyses and significance testing were not applied to these variables. All analyses were performed using R (version 4.3.3; GNU General Public License; R Core Team 2024).

## 3 Results

### 3.1 Changes in the Physical Traits of Mungbean Sprouts Treated With Micronutrients

**FIGURE 1:**
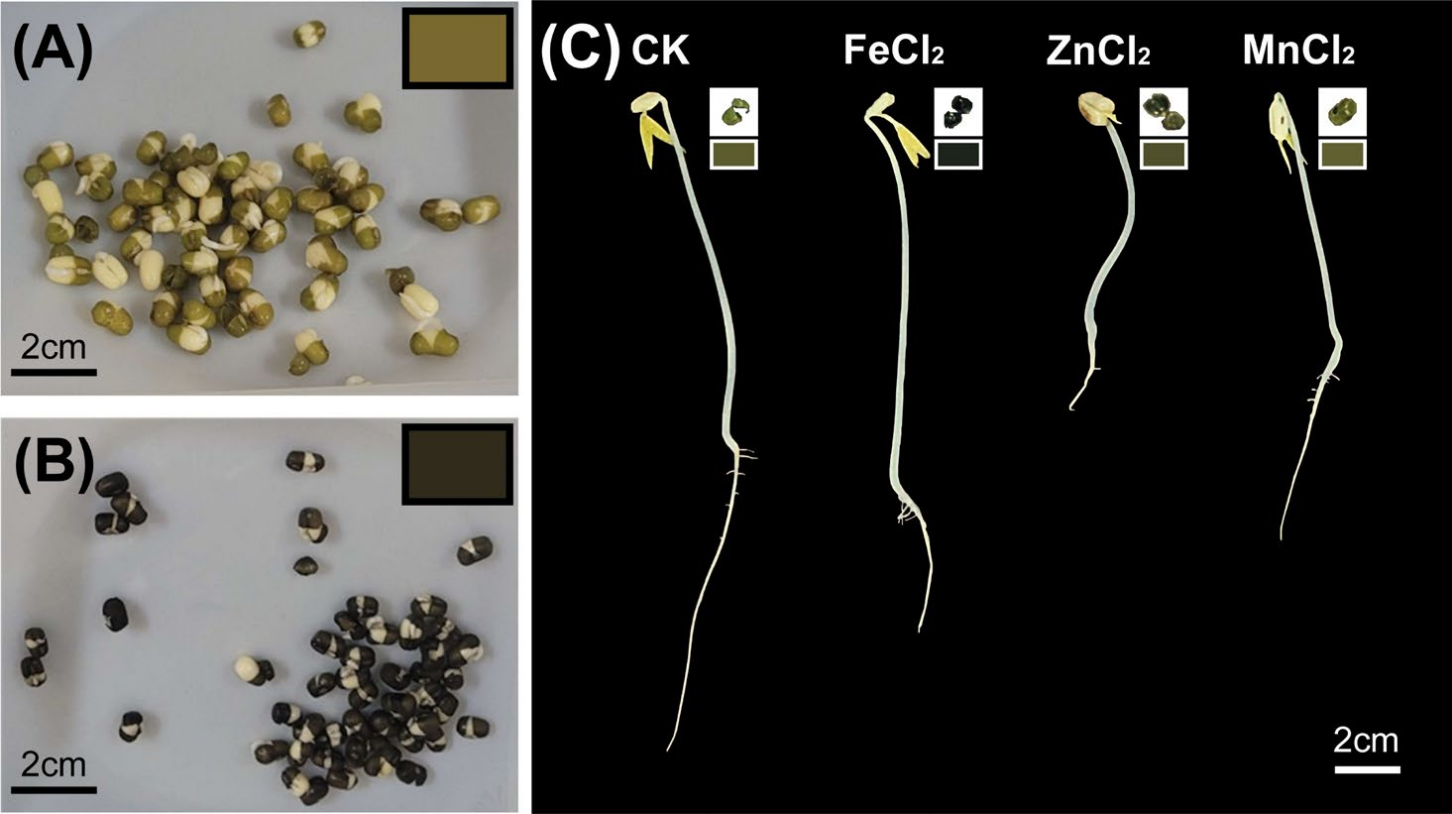
Morphological traits of mungbean seeds and sprouts after mineral treatments. (A) Mungbean seeds soaked in distilled water. (B) Mungbean seeds soaked in 5 mM FeCl_2_. (C) Representative images of mungbean sprouts cultivated for 3 days after mineral treatments. Colored boxes indicate seed coat colors. Scale bar = 2 cm.

When mungbean seeds were soaked in 5 mM solutions of FeCl_2_, MnCl_2_, and ZnCl_2_ for 17 h, FeCl_2_‐treated seeds developed a dark brown seed coat (R: 48, G: 43, B: 26). In contrast, MnCl_2_‐ and ZnCl_2_‐treated seeds showed no color difference compared with distilled water control (CK) seeds (R: 121, G: 105, B: 47), but their growth was noticeably inhibited relative to CK (Figure 1A,B; Figure S2).

To further examine the effect of Fe ions, FeCl_2_‐soaked seeds were cultivated into sprouts for 3 days (Fe‐72 h) (Figure 1C).

**FIGURE 2:**
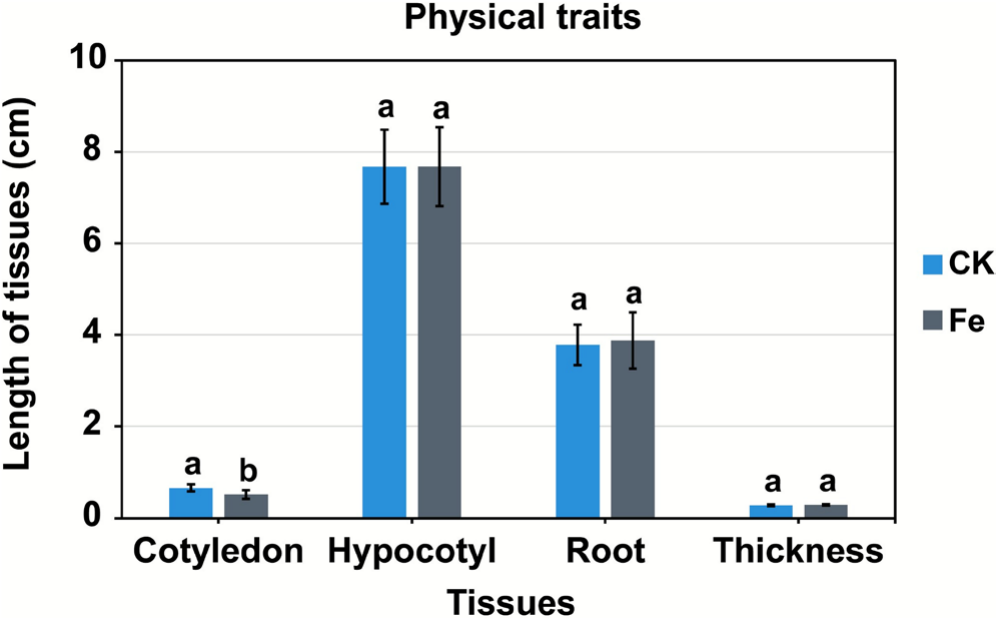
Physical trait changes in mungbean sprouts induced by mineral treatments. Blue and gray indicate the control (CK) and FeCl_2_ treatment (Fe), respectively. Quantitative measurements were obtained from 50 individual sprouts per treatment. Error bars indicate standard deviation (SD). Different lowercase letters denote statistical significance (*p* < 0.05).

**FIGURE 3:**
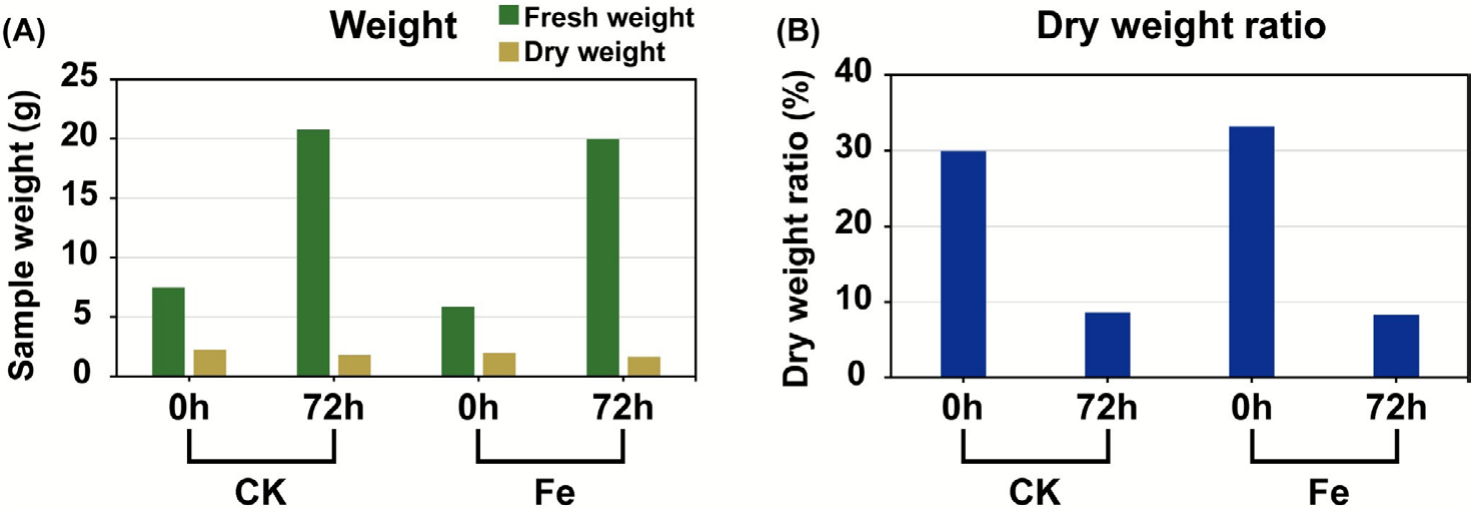
Changes in the weight of mungbean sprouts after FeCl_2_ treatment. (A) Fresh and dry weights of mungbean sprouts measured from a single pooled sample composed of 50 sprouts per treatment, presented to compare overall treatment‐dependent trends. Green indicates fresh weight, and yellowish brown represents dry weight. (B) Fresh‐to‐dry weight ratio of mungbean sprouts.

The lengths and thicknesses of the hypocotyl and root did not differ significantly between Fe‐72 h and sprouts treated with distilled water (CK‐72 h) (Figure 2). Fresh weights were 19.98 and 20.78 g for Fe‐72 h and CK‐72 h, whereas dry weights were 1.66 and 1.78 g, respectively. The fresh‐to‐dry weight ratios were 8.29% (Fe‐72 h) and 8.57% (CK‐72 h), indicating no notable growth differences between Fe and CK (Figure 3A,B).

### 3.2 Changes in Total Phenolic and Flavonoid Contents and Antioxidant Activity by Micronutrient Treatment

**FIGURE 4:**
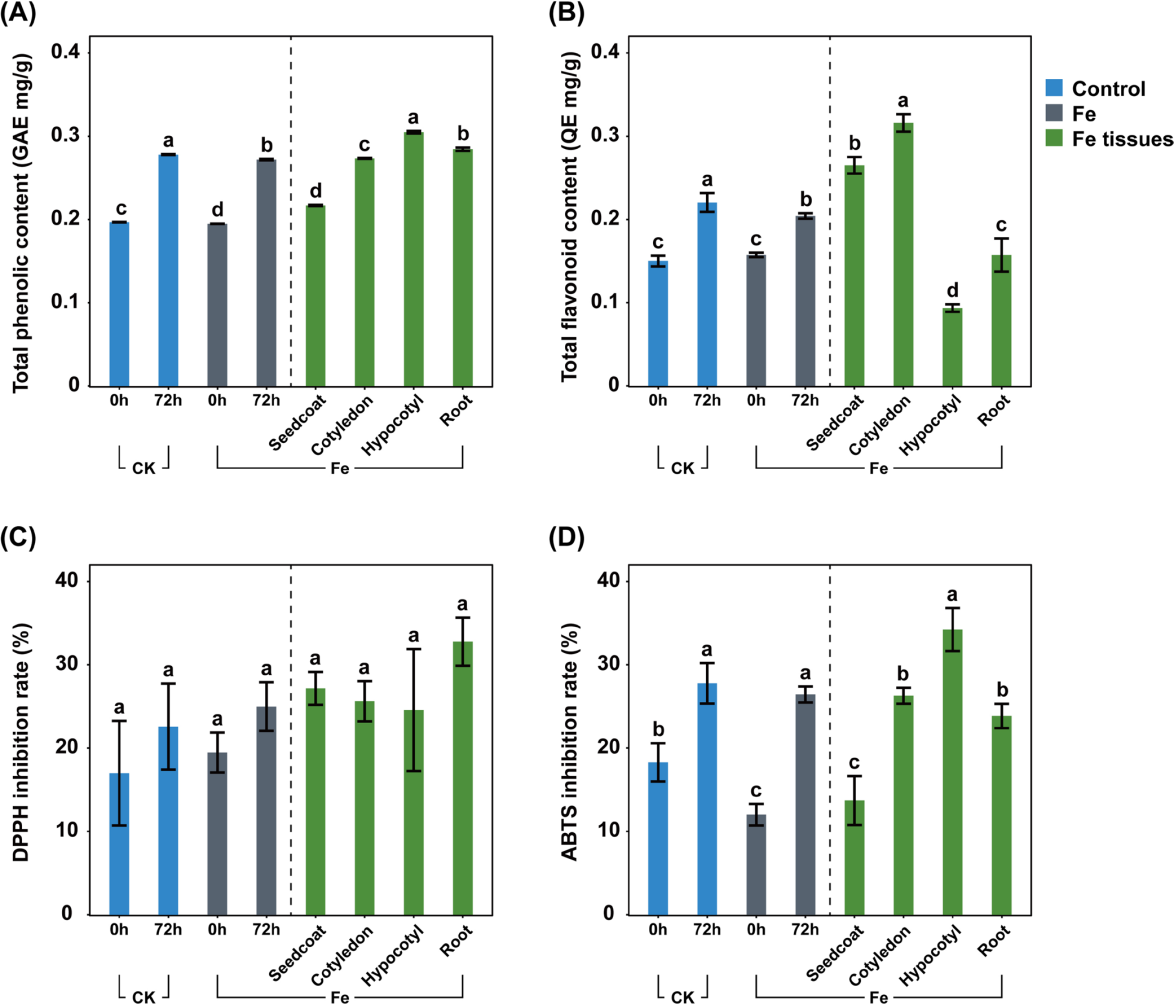
Comparison of the biochemical compounds and antioxidant activity in mungbean sprouts after FeCl_2_ treatment. (A) Total phenolic content. (B) Total flavonoid content. (C) DPPH radical scavenging activity. (D) ABTS radical scavenging activity. Blue, gray, and green represent CK, Fe, and Fe‐treated tissues, respectively. Error bars indicate SD. Lowercase letters denote statistical significance (*p* < 0.05).

Total phenolic and flavonoid contents increased after 72 h compared with 0 h in both CK and Fe groups (Figure 4A,B). At 72 h, total phenolics were 0.28 ± 0.00 mg/g in CK and 0.27 ± 0.00 mg/g in Fe. Total flavonoids were 0.22 ± 0.01 mg/g in CK and 0.20 ± 0.00 mg/g in Fe.

Tissue‐specific analysis of Fe‐72 h sprouts revealed that the hypocotyl had the highest phenolic content 0.3 ± 0.00 mg/g, whereas the seed coat had the lowest (0.22 ± 0.00 mg/g). For flavonoids, the cotyledon had the highest content (0.32 ± 0.01 mg/g), followed by the seed coat (0.27 ± 0.01 mg/g), whereas the hypocotyl showed the lowest (0.09 ± 0.00 mg/g).

For DPPH radical scavenging activity, CK and Fe showed no significant difference at 0 h (16.99% ± 6.28% and 19.46% ± 2.39%) or at 72 h (22.58% ± 5.17% and 24.98% ± 2.92%, respectively) (Figure 4C).

For ABTS radical scavenging activity, CK showed significantly higher antioxidant activity than Fe at 0 h (18.28% ± 2.30% and 12.00% ± 1.29%) (Figure 4D). However, after 72 h of cultivation, no significant difference was observed, with CK at 27.78% ± 2.43% and Fe at 26.44% ± 0.96%. For antioxidant activity, there was no significant difference in DPPH radical scavenging activity among the tissues. For ABTS radical scavenging activity, the hypocotyl showed the highest value at 34.23% ± 2.58%, whereas the seed coat had the lowest value at 13.71% ± 2.94%.

### 3.3 Changes in the Contents of Secondary Metabolites by Micronutrient Treatment

**FIGURE 5:**
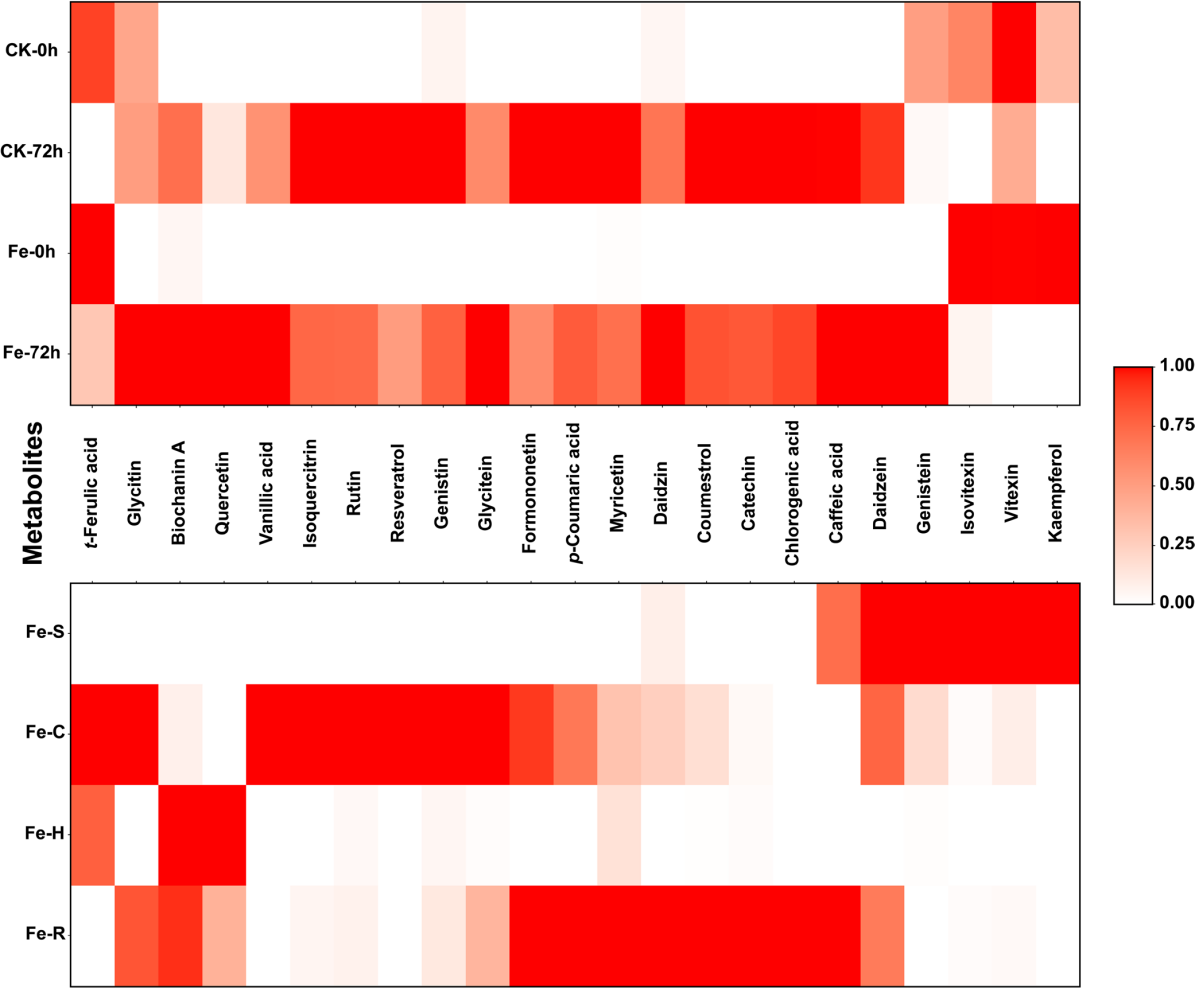
Heatmap of secondary metabolite content in mungbean sprouts after FeCl_2_ treatment. Relative amounts of secondary metabolites are represented on a color scale from white to red. Fe–S, C, H, and R indicate the seed coat, cotyledon, hypocotyl, and root of Fe‐treated sprouts, respectively. Statistical significance of differences in secondary metabolite contents among treatments and tissues was assessed using Duncan's multiple range test (*p* < 0.05).

Among the 31 standard substances analyzed using UPLC, 23 compounds were detected in mungbean sprout extracts. To confirm that the analyzed compounds were indeed the major constituents in mungbean sprout extracts, a representative HPLC chromatogram is provided in Figure S3. Both CK and Fe groups showed significantly higher levels of *t*‐ferulic acid, isovitexin, kaempferol, and vitexin at 0 h compared with 72 h (Figure 5). At 0 h, Fe‐treated sprouts contained significantly higher amounts of isovitexin (295.91 ± 2.68 mg/100 g) and kaempferol (2.25 ± 1.02 mg/100 g) than CK (198.13 ± 5.00 and 0.77 ± 0.10 mg/100 g, respectively).

At CK‐72 h, CK sprouts had significantly higher levels of catechin, *p*‐coumaric acid, coumestrol, formononetin, isoquercitrin, myricetin, resveratrol, and rutin compared with Fe‐72 h sprouts. Conversely, Fe‐72 h sprouts showed significantly higher levels of biochanin A, daidzein, daidzin, genistein, glycitein, glycitin, quercetin, and vanillic acid compared with CK‐72 h.

In CK, genistein content was significantly higher at 0 h (6.02 ± 0.22 mg/100 g) than at 72 h (2.86 ± 0.11 mg/100 g), whereas glycitin showed no significant change between 0 h (31.64 ± 0.40 mg/100 g) and 72 h (31.86 ± 0.20 mg/100 g). In Fe, genistein content increased significantly at 72 h (9.40 ± 0.17 mg/100 g) compared with 0 h (2.65 ± 0.13 mg/100 g), and glycitin content also increased significantly at 72 h (34.24 ± 0.07 mg/100 g) relative to 0 h (29.44 ± 0.30 mg/100 g).

Tissue‐specific analysis of Fe‐72 h sprouts revealed distinct metabolite distributions. The seed coat contained the highest levels of daidzein, genistein, isovitexin, kaempferol, and vitexin. The cotyledon showed the highest levels of *t*‐ferulic acid, genistin, glycitein, glycitin, isoquercitrin, resveratrol, rutin, and vanillic acid. Quercetin was most abundant in the hypocotyl, whereas the root contained the highest levels of caffeic acid, catechin, *p*‐coumaric acid, coumestrol, chlorogenic acid, daidzin, formononetin, and myricetin. Notably, daidzein, daidzin, and genistin were detected in all Fe tissues; kaempferol was specific to the seed coat, resveratrol and vanillic acid were specific to the cotyledon, and chlorogenic acid was detected only in the root.

## 4 Discussion

Legume crops are important food sources worldwide because of their high nutrient content, such as protein and carbohydrate, and their high yield. Isoflavones such as daidzein, genistein, and glycitein are major secondary metabolites in legumes and act as phytoestrogens in humans, effectively preventing osteoporosis and breast cancer (Hou et al. 2019). Mungbean, a major legume crop, contains various secondary metabolites, whose levels increase when the seeds are cultivated as sprouts (An et al. 2020; Kartikeyan et al. 2022; Peng et al. 2008). Environmental stress is a critical challenge to crop cultivation, and various strategies are explored to reduce stress‐induced damage. Mineral treatments, in particular, can enhance plant resistance to stress and increase secondary metabolite content (Iqbal et al. 2020; Rotaru 2011). In this study, we investigated the effects of FeCl_2_ treatment on mungbean sprout cultivation, focusing on both physical traits and changes in secondary metabolite content.

The seed coat color of mungbeans treated with ZnCl_2_ and MnCl_2_ was yellowish‐brown, similar to that of the CK, whereas FeCl_2_ treatment resulted in a dark brown seed coat (Figure 1). Tannins, a class of polyphenolic compounds, are present in both the cotyledons and seed coats of mungbean, but are predominantly concentrated in the seed coats (Das et al. 2020; Hou et al. 2019). In legumes, seed coat coloration is largely attributed to variation in tannin content, highlighting the seed coat as the major site of tannin accumulation (Kakati et al. 2010; Sudhakaran et al. 2024). Notably, tannins, including tannic acid, interact with Fe^2+^ and Fe^3+^ ions to form dark‐colored complexes (Fu and Chen 2019). Therefore, the dark brown coloration observed in FeCl_2_‐treated mungbean seed coats may result from the formation of iron–tannin complexes during soaking. However, the precise mechanism underlying this color change remains unclear, and further studies are required to clarify the specific role of tannins in this process.

In alfalfa ( *Medicago sativa* L.), supplying Fe through iron chelate enhances nutritional value and secondary metabolite content in sprouts (Park et al. 2014). In this study, CK‐72 h sprouts showed significantly higher total phenolic and flavonoid contents compared with Fe‐72 h (Figure 4A,B). UPLC analysis revealed that most flavonoids were significantly higher in CK‐72 h than in Fe‐72 h, whereas isoflavones, including daidzein, genistein, and glycitein, were significantly higher in Fe‐72 h (Figure 5). Notably, genistein content decreased over time in CK but increased in Fe‐treated sprouts during cultivation.

Metal chelates are complexes of metal ions with substances such as amino acids or organic acids, enhancing plant absorption. Isoflavones, as major secondary metabolites of legumes, are potent iron chelators (Toscano and Russo 2016).

In the Fe‐72 h treatment, total phenolic and flavonoid contents were lower than those in the control (CK), whereas isoflavone content was significantly increased. Previous studies have shown that under conditions of metal ion exposure or oxidative stress, plant secondary metabolic pathways are not activated indiscriminately; rather, the biosynthesis of specific metabolites that play important roles in stress adaptation can be selectively induced (Muthusamy and Lee 2024; Trush and Pal'ove‐Balang 2023). Considering these findings, the results observed in the present study suggest that FeCl_2_ treatment did not promote the overall accumulation of phenolic compounds, but instead was associated with a selective metabolic response in which the accumulation of specific metabolites, such as isoflavones that are known to play important roles in metal‐ and redox‐related stress responses, was preferentially enhanced.

In legume crops, isoflavones are well recognized as representative secondary metabolites involved in defense responses under environmental stress conditions and are known to exert antioxidant functions by scavenging reactive oxygen species (ROS), thereby mitigating oxidative damage. Indeed, previous studies in legumes have reported that the antioxidant activity of isoflavones is associated with enhanced tolerance to various abiotic stresses, and studies in soybean have further demonstrated that increased isoflavone content is correlated with improved tolerance to drought stress (Akitha Devi and Giridhar 2015; Tian et al. 2014). Therefore, the increase in isoflavone content observed following Fe treatment in this study may be interpreted as an adaptive metabolic response to physiological stress that can occur during mungbean sprout cultivation.

However, because the present study did not investigate gene expression related to isoflavone biosynthesis, enzyme activities, or direct interactions between metal ions and isoflavones, this interpretation is based on metabolite‐level observations. Further molecular and biochemical studies will be required to elucidate the regulatory mechanisms underlying Fe‐induced modulation of isoflavone accumulation in mungbean sprouts.

Although plant responses to mineral treatments are generally concentration‐dependent, the concentration applied in this study allowed the evaluation of mineral‐induced physiological and metabolic responses without inducing severe phytotoxic stress. Further studies incorporating concentration‐gradient experiments will be required to more systematically investigate dose‐dependent effects and to refine optimal mineral treatment conditions.

The results of secondary metabolite measurements in various Fe‐treated tissues showed that the major iron chelators, isoflavone aglycones (daidzein, genistein, and glycitein), were relatively higher in the seed coat and cotyledons (Toscano and Russo 2016). We hypothesize that these tissues exhibited the highest accumulation of isoflavone aglycones because they were directly exposed to the FeCl_2_ solution during soaking, whereas the roots, which also developed during soaking, showed the next highest accumulation. The hypocotyl, which develops during cultivation after soaking, showed relatively lower isoflavone aglycone content, likely because of a lack of direct contact with iron ions. These findings suggest that tissues directly exposed to minerals undergo significant metabolic changes compared with those indirectly affected, and that the timing of tissue development during mineral treatment can influence the content of specific secondary metabolites.

Beyond the physiological and metabolic implications discussed above, the selective increase in isoflavone aglycones observed in FeCl_2_‐treated mungbean sprouts may also have practical significance for food quality. Isoflavone aglycones are known to exhibit higher bioavailability and well‐established health benefits; therefore, their preferential accumulation could enhance the nutritional and functional value of mungbean sprouts. Although FeCl_2_ treatment resulted in a darker seed coat color, this visual change did not adversely affect sprout growth or other physical characteristics, suggesting that the treatment does not compromise overall product quality. Moreover, the FeCl_2_ concentration applied in this study was relatively low, and no abnormal growth or phytotoxic effects were observed under the experimental conditions. Nevertheless, further studies assessing sensory attributes, consumer acceptance, and iron accumulation in edible tissues are required to fully evaluate the applicability of this approach in relation to food safety regulations.

## 5 Conclusion

This study investigated the physical and metabolomic changes in mungbean sprouts induced by FeCl_2_ treatment, which altered seed coat color. No significant differences in physical traits were observed between CK‐72 h and Fe‐72 h. Although total phenolic and flavonoid contents were higher in CK than in Fe, the isoflavone content was significantly higher in Fe‐72 h. These results indicate that FeCl_2_ treatment does not affect the physical quality of mungbean sprouts but can enhance the metabolic quality by increasing isoflavone content.

## Author Contributions

A.P.: Data curation; normal analysis; validation; visualization; writing original draft; and writing – review editing. B.C.K.: Data curation; formal analysis; investigation; software; visualization; methodology; and writing – original draft. S.D.L: Resources and writing – review and editing. S.H.P.: Supervision; funding acquisition; and writing – review and editing. J.H.: Conceptualization; resources; supervision; funding acquisition; methodology; writing – review and editing; and project administration.

## Funding

This work was supported by the Ministry of Education, 2025‐RISE‐10‐004. Rural Development Administration, RS‐2024‐00398371.

## Ethics Statement

This study did not involve human or animal testing.

## Consent

The authors have nothing to report.

## Conflicts of Interest

The authors declare no conflicts of interest.

## Supporting information


**Figure S1:** HPLC calibration curves of standard compounds. Linear relationships between concentration and peak area are presented, with regression equations, coefficients of determination (*R* ^2^), and the limits of detection (LOD) and quantification (LOQ) for each compound.
**Figure S2:** Morphological traits of soaked mungbean seeds after mineral treatments. (A) Mungbean seeds soaked in 5 mM ZnCl_2_. (B) Mungbean seeds soaked in 5 mM MnCl_2_. Colored boxes indicate seed coat colors. Scale bar = 2 cm.
**Figure S3:** Representative HPLC chromatogram of mungbean sprout extracts recorded at 280 nm, selected to enhance the visualization of major phenolic compounds and isoflavones. Peaks corresponding to the major compounds quantified in this study are indicated.

## Data Availability

Data available in the article Supporting Information.
