# Supplementary material for: Impact of Iron (II) Chloride Treatment on the Physical and Metabolic Changes in Mungbean Sprouts
Source: Food Sci Nutr. 2026 Feb 24;14(3):e71558. doi: 10.1002/fsn3.71558 (PMC12930292; doi:10.1002/fsn3.71558)
Supplement: Supplementary file 1 — Figure S1: HPLC calibration curves of standard compounds. Linear relationships between concentration and peak area are presented, with regression equations, coefficients of determination (R 2), and the limits of detection (LOD) and quantification (LOQ) for each compound. Figure S2: Morphological traits of soaked mungbean seeds after mineral treatments. (A) Mungbean seeds soaked in 5 mM ZnCl2. (B) Mungbean seeds soaked in 5 mM MnCl2. Colored boxes indicate seed coat colors. Scale bar = 2 cm. Figure S3: Representative HPLC chromatogram of mungbean sprout extracts recorded at 280 nm, selected to enhance the visualization of major phenolic compounds and isoflavones. Peaks corresponding to the major compounds quantified in this study are indicated. [file FSN3-14-e71558-s001.pdf]

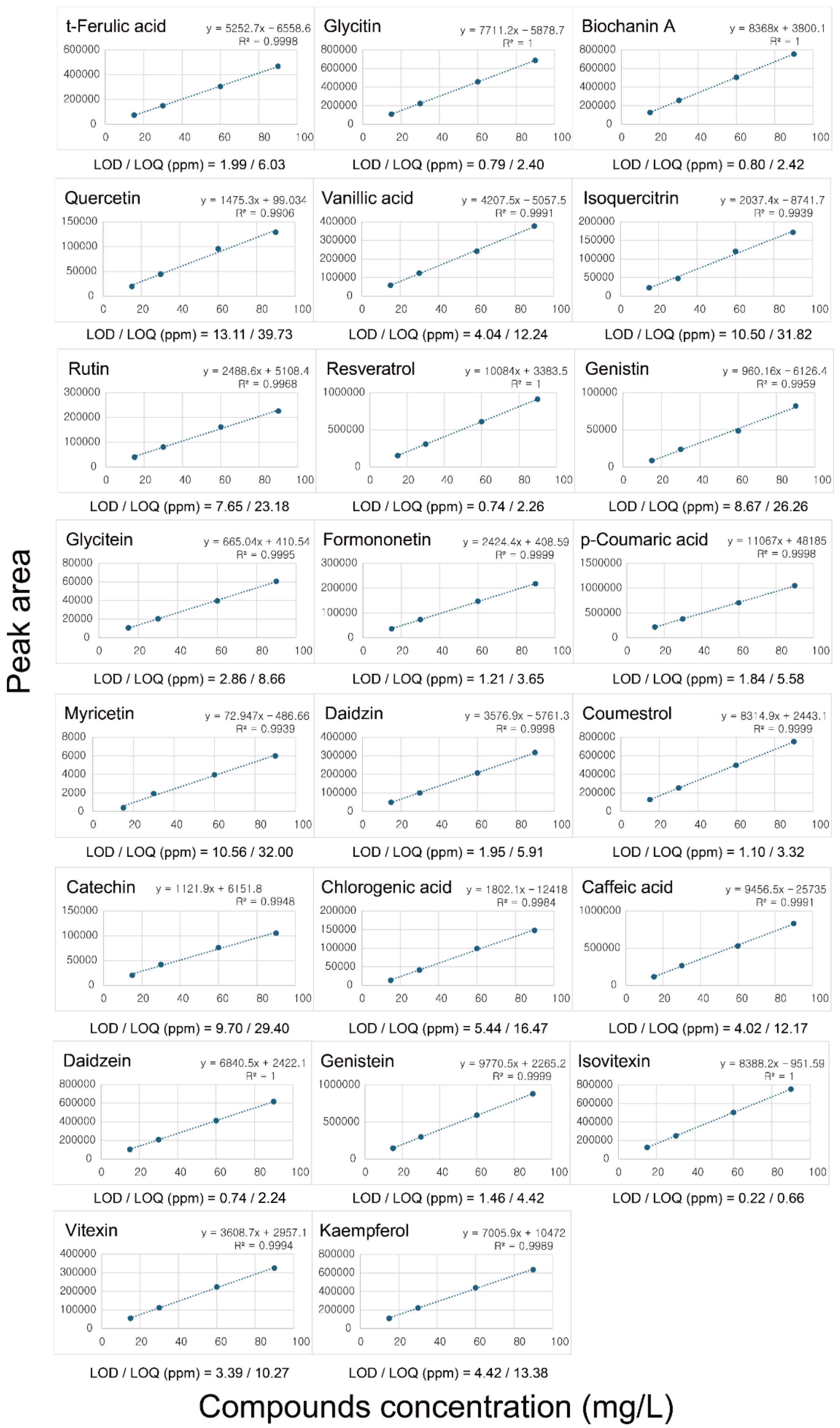

t-Ferulic acid
y = 5252.7x − 6558.6
R² = 0.9998
LOD / LOQ (ppm) = 1.99 / 6.03
Glycitin
y = 7711.2x − 5878.7
R² = 1
LOD / LOQ (ppm) = 0.79 / 2.40
Biochanin A
y = 8368x + 3800.1
R² = 1
LOD / LOQ (ppm) = 0.80 / 2.42
Quercetin
y = 1475.3x + 99.034
R² = 0.9906
LOD / LOQ (ppm) = 13.11 / 39.73
Vanillic acid
y = 4207.5x − 5057.5
R² = 0.9991
LOD / LOQ (ppm) = 4.04 / 12.24
Isoquercitrin
y = 2037.4x − 8741.7
R² = 0.9939
LOD / LOQ (ppm) = 10.50 / 31.82
Rutin
y = 2488.6x + 5108.4
R² = 0.9968
LOD / LOQ (ppm) = 7.65 / 23.18
Resveratrol
y = 10084x + 3383.5
R² = 1
LOD / LOQ (ppm) = 0.74 / 2.26
Genistin
y = 960.16x − 6126.4
R² = 0.9959
LOD / LOQ (ppm) = 8.67 / 26.26
Glycitein
y = 665.04x + 410.54
R² = 0.9995
LOD / LOQ (ppm) = 2.86 / 8.66
Formononetin
y = 2424.4x + 408.59
R² = 0.9999
LOD / LOQ (ppm) = 1.21 / 3.65
p-Coumaric acid
y = 11067x + 48185
R² = 0.9998
LOD / LOQ (ppm) = 1.84 / 5.58
Myricetin
y = 72.947x − 486.66
R² = 0.9939
LOD / LOQ (ppm) = 10.56 / 32.00
Daidzin
y = 3576.9x − 5761.3
R² = 0.9998
LOD / LOQ (ppm) = 1.95 / 5.91
Coumestrol
y = 8314.9x + 2443.1
R² = 0.9999
LOD / LOQ (ppm) = 1.10 / 3.32
Catechin
y = 1121.9x + 6151.8
R² = 0.9948
LOD / LOQ (ppm) = 9.70 / 29.40
Chlorogenic acid
y = 1802.1x − 12418
R² = 0.9984
LOD / LOQ (ppm) = 5.44 / 16.47
Caffeic acid
y = 9456.5x − 25735
R² = 0.9991
LOD / LOQ (ppm) = 4.02 / 12.17
Daidzein
y = 6840.5x + 2422.1
R² = 1
LOD / LOQ (ppm) = 0.74 / 2.24
Genistein
y = 9770.5x + 2265.2
R² = 0.9999
LOD / LOQ (ppm) = 1.46 / 4.42
Isovitexin
y = 8388.2x − 951.59
R² = 1
LOD / LOQ (ppm) = 0.22 / 0.66
Vitexin
y = 3608.7x + 2957.1
R² = 0.9994
LOD / LOQ (ppm) = 3.39 / 10.27
Kaempferol
y = 7005.9x + 10472
R² = 0.9989
LOD / LOQ (ppm) = 4.42 / 13.38
Peak area
Compounds concentration (mg/L)

Supplementary figure 1. HPLC calibration curves of standard compounds. Linear relationships between concentration and peak area are presented, with regression equations, coefficients of determination (R^2), and the limits of detection (LOD) and quantification (LOQ) for each compound.

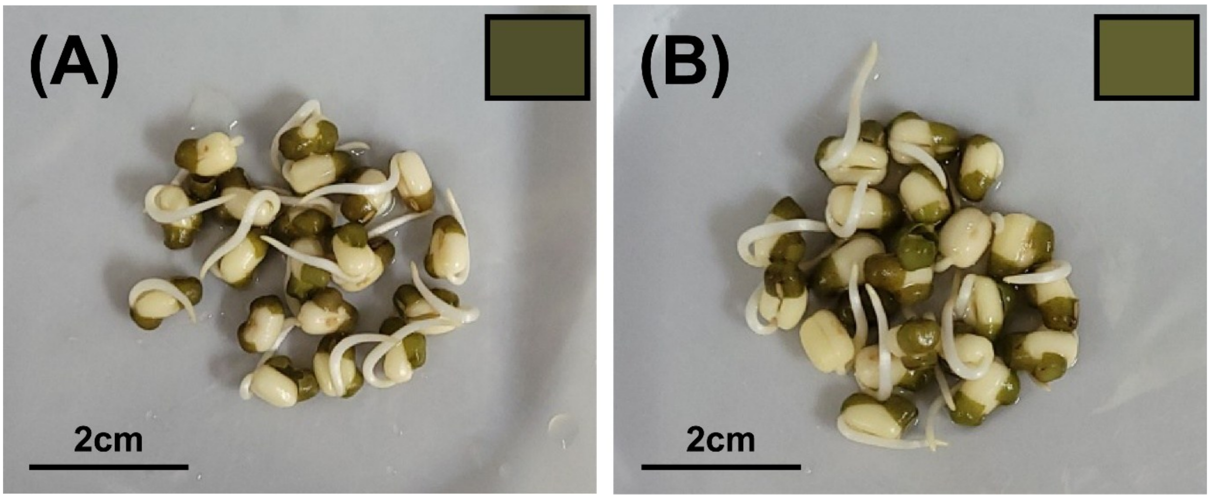

Supplementary figure 2. Morphological traits of soaked mungbean seeds after mineral treatments. (A) Mungbean seeds soaked in 5 mM $ZnCl_2$. (B) Mungbean seeds soaked in 5 mM $MnCl_2$. Colored boxes indicate seed coat colors. Scale bar = 2 cm.

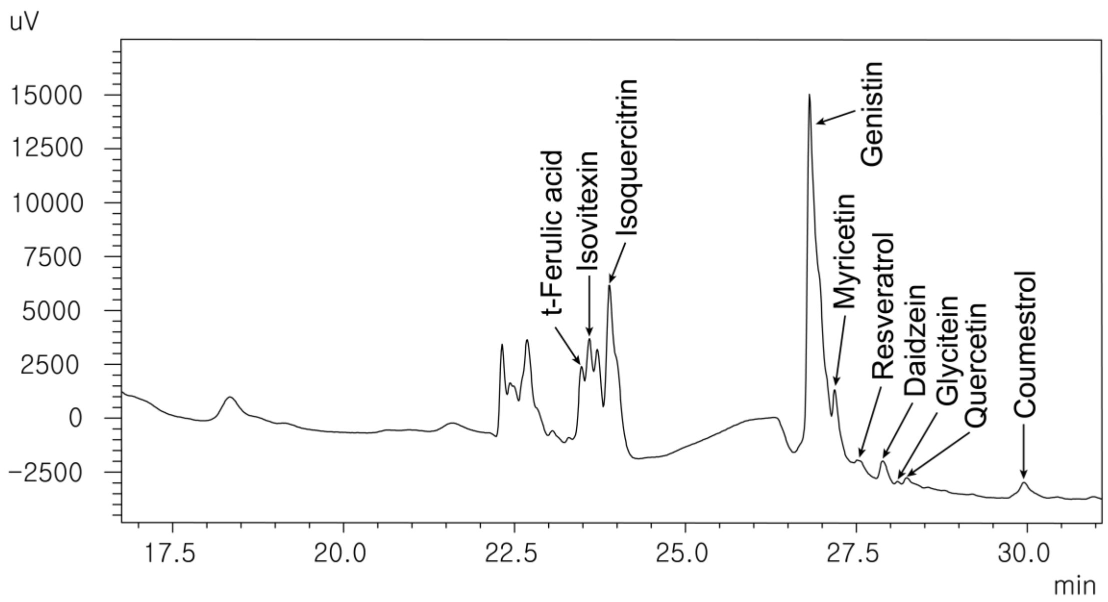

Supplementary figure 3. Representative HPLC chromatogram of mungbean sprout extracts recorded at 280 nm, selected to enhance the visualization of major phenolic compounds and isoflavones. Peaks corresponding to the major compounds quantified in this study are indicated.
